# Integrating a Convolutional Neural Network and MultiHead Attention with Long Short-Term Memory for Real-Time Control During Drying: A Case Study of Yuba (*Tofu Skin*)

**DOI:** 10.3390/foods15020245

**Published:** 2026-01-09

**Authors:** Jiale Guo, Jie Wu, Lixuan Zhang, Ziqin Peng, Lixuan Wei, Wuxia Li, Jingzhi Shen, Yanhong Liu

**Affiliations:** 1 College of Engineering, China Agricultural University, 17 Qinghua Donglu, Beijing 100083, China; guojlcau@163.com (J.G.);; 2 YanTai Research Institute, China Agricultural University, Binhai Middle Road, Yantai 264670, China

**Keywords:** Long Short-Term Memory, Convolutional Neural Network, MultiHead Attention, hot-air drying, real-time control

## Abstract

Achieving comprehensive improvements in the drying rate (DR) and the quality after drying of agricultural products is a major goal in the field of drying. To further shorten the drying time while improving product quality, this study introduced a Convolutional Neural Network (CNN) and MultiHead Attention (MHA) to enhance the prediction accuracy of the Long Short-Term Memory (LSTM) network regarding the properties of dried samples. These properties included DR, shrinkage rate (SR), and total color difference (Δ*E*). The CNN-LSTM-MHA network was proposed, developing a novel hot-air drying (HAD) scenario utilizing an intelligent temperature control system based on the real dynamics of material properties. The results of drying experiments with temperature-sensitive yuba showed that the CNN-LSTM-MHA network’s predictive accuracy was better than that of other networks, as evidenced by its coefficient of determination (R^2^: 0.9855–0.9999), root mean square error (RMSE: 0.0001–0.0099), and mean absolute error (MAE: 0.0001–0.0120). Comparative analysis with fixed-temperature drying indicated that CNN-LSTM-MHA-controlled drying significantly reduced drying time and enhanced the SR, color, rehydration ratio (RR), texture, protein content, fat content, and microstructure of yuba. Overall, the findings highlight the potential of CNN-LSTM-MHA-based intelligent drying as a viable strategy for yuba stick processing, providing insights for other food drying applications.

## 1. Introduction

Drying is a crucial method for extending the shelf life and preventing mildew growth on agricultural products. However, the long-term exposure of agricultural products to high-temperature environments during drying affects their quality [1]. To solve this problem, researchers have developed various techniques, such as intermittent grain drying with tempering and temperature control during different drying stages of fruits and vegetables [2,3]. However, these methods only set different drying temperatures in several stages, which is insufficient for complex drying systems.

Real-time drying temperature control (RTDTC) technology can improve the quality of dried products while reducing drying time. For instance, Vilas et al. [4] applied RTDTC technology during freeze drying, and their results showed that RTDTC reduced the drying time by about 30% compared with the standard strategy. In the study of Nadian et al. [5], the authors predicted the drying time, color, and shrinkage rate (SR) of kiwifruit slices under a microwave–hot air drying process based on an Artificial Neural Network (ANN) model. Their results showed that the drying time was shortened by 40%, while the total color difference (Δ*E*) was reduced by 73.42%. However, the inherent characteristics of the model they applied in this study led to difficulties in establishing a correlation between quality and drying time, which limited the prediction accuracy of the model, as drying is a process in which a product’s state changes over time [6]. Therefore, applying a prediction model that can capture the time series relationship within the data is expected to enhance the accuracy of model predictions.

The Long Short-Term Memory (LSTM) network is particularly adept at capturing the temporal dynamics inherent in the input data [7]. In Jia et al.’s [6] study on the drying kinetics and color prediction of apple slices, they confirmed that the LSTM network achieved the highest accuracy (coefficient of determination (R^2^) > 0.98). However, it is regrettable that their research did not integrate the predicted results into the dryer for the real-time control of the drying temperature, which also limited further optimization of the drying process. Guo et al. [8] utilized the LSTM model for the real-time control of drying parameters (temperature, humidity, and air velocity) during the HAD of *Pleurotus eryngii*. Their results showed that the composite scores of the samples after real-time control of the drying parameters were 6% higher than that of those with the highest score in the full-factorial experiment [8]. However, they found that when the drying parameters were frequently changed, the LSTM’s prediction accuracy was significantly decreased. Therefore, it is necessary to optimize the LSTM further in order to improve its prediction accuracy in situations involving frequent changes in drying parameters. In addition, to test the effectiveness of the real-time control system based on the improved LSTM, temperature-sensitive samples are more applicable for testing.

Yuba is a sensitive product to drying temperatures. It is a film that forms on the surface of soybean milk when boiled, and is usually dried before being transported and sold [9]. Therefore, using yuba for drying experiments can intuitively demonstrate the effectiveness of the real-time temperature control system based on the improved LSTM.

Therefore, the purposes of this study were to (1) improve the structure of LSTM and test the prediction accuracy of the network and (2) explore the effects of different drying temperature control methods on drying kinetics and qualities. Compared with our previous research [8], this work proposes a CNN-LSTM-MHA model which addresses the accuracy loss of LSTM under parameter frequency fluctuations by integrating local feature extraction and multi-dimensional attention.

## 2. Materials and Methods

### 2.1. Raw Materials

**Figure 1:**
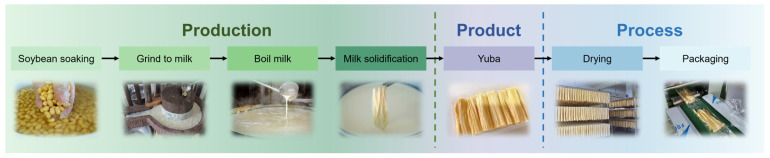
Diagram of yuba processing.

The yuba (the production method of which is shown in Figure 1) was purchased from a local supermarket in Haidian District, Beijing, China, and stored in thermally sealed polyethylene bags at 4 °C for no longer than one week before use. Before drying, the samples were cut to a length of 60.00 ± 4.87 mm. The initial moisture content of the fresh samples was measured to be 56.64% ± 0.89% on a wet basis (w.b.).

### 2.2. Experimental Equipment and Design

The HAD system used to conduct the drying experiments on yuba was fully described in our previous study [8]. The system was designed and established in our lab for the online monitoring of the appearance qualities and DR of agricultural products in order to control the temperature, humidity, and air velocity during drying [8]. To ensure precise data acquisition and control of drying parameters throughout the experiment, an SHT30 temperature and humidity sensor (Sensirion, Frauenfeld, Switzerland) was employed. This sensor offers a temperature accuracy of ±0.3 °C and a humidity accuracy of ±3% RH. Additionally, a portable air velocity meter (HT9829, Xinsite, Dongguan, China) with a precision of ±5% was utilized to measure air velocity, ensuring accurate pulse width modulation (PWM) regulation. At the top of the drying chamber, a Raspberry PI 4B, an industrial camera (DF200, Jieruiweitong, Shenzhen, China), and a ring shadowless lamp (R-90, Topvision, Shenzhen, China) were mounted. The system also included a gas heating and circulation unit, comprising a heater, air ducts, and a centrifugal fan, to regulate air velocity and temperature. The centrifugal fan was linked to an exhaust pipe, which directed the expelled gas into the drying chamber. Furthermore, a weight sensor was integrated into the drying chamber to enable the real-time monitoring of sample weight. A water tank and humidification system were also incorporated to adjust the relative humidity of the hot air.

Before drying, the yuba sticks were placed evenly on three trays, with twelve sticks for each tray. One yuba stick was placed in the upper tray for appearance quality tracking. The drying air velocity was set at 1.50 ± 0.20 m/s. During drying, images of the sample on the upper plate were taken every 1 min until a final moisture content (w.b.) of 10% (drying endpoint) was reached. The design of specific experimental parameters is shown in Table 1.

### 2.3. The Real-Time Control Strategy of Drying Temperature

#### 2.3.1. LSTM Model

**Figure 2:**
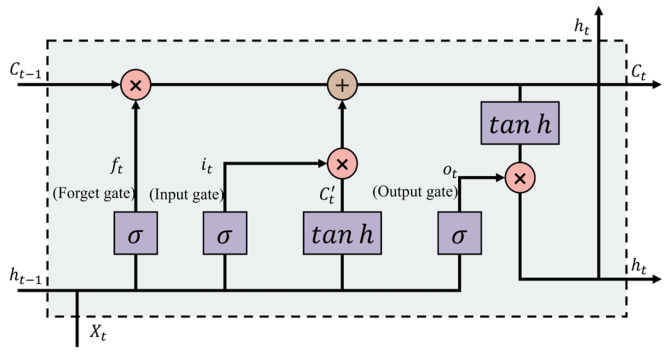
Diagram of the unit structure of the LSTM.

The LSTM model, a widely utilized variant of Recurrent Neural Networks (RNNs), excels in handling long-term dependencies, thereby improving the network’s capacity to model temporal dynamics in sequential data [10]. This advantage is attributed to the specialized architecture of LSTM cells, which incorporate three gate mechanisms, as depicted in Figure 2. The recurrent nature of LSTM allows for sequential data processing and iterative updates of internal states at every time step, enabling the network to effectively learn temporal patterns in time series data. The memory cell in LSTM is governed by three gate units: (1) the function of the input gate is to determine which information from the current time step’s input should be saved to the cell state; (2) the function of the forget gate is to decide which information from the previous time step’s cell state should be discarded; and (3) the function of the output gate is to control which parts of the cell state need to be output as the current output value. Specifically, after receiving the data, the forget gate decides which information to keep from the previous time step, and the input gate updates the current time step’s input into the state, generating a candidate state. Then, the cell state is updated by means of element-wise multiplication with the outputs of the forget gate and the input gate. Finally, the output gate controls the hidden state output for the current time step and, together with the updated cell state, determines the final output result of the LSTM, as described in Equations (1)–(6) [8]. The interplay of these gates, combined with the application of tanh and sigmoid activation functions, mitigates the issue of gradient decay during backpropagation.

(1) $$f_{t}=\sigma \left(W_{f}\cdot \left[h_{t-1},X_{t}\right]+b_{f}\right)$$

(2) $$i_{t}=\sigma \left(W_{i}\cdot \left[h_{t-1},X_{t}\right]+b_{i}\right)$$

(3) $$C_{t}^{\prime }=\tan h\left(W_{C}\cdot \left[h_{t-1},X_{t}\right]+b_{C}\right)$$

(4) $$C_{t}=f_{t}C_{t-1}+i_{t}C_{t}^{\prime }$$

(5) $$o_{t}=\sigma \left(W_{o}\cdot \left[h_{t-1},X_{t}\right]+b_{o}\right)$$

(6) $$h_{t}=o_{t}\tan h\left(C_{t}\right)$$

where $f_{t}$, $i_{t}$, $C_{t}^{\prime }$, $o_{t}$, $C_{t}$, and $h_{t}$ are the forget gate, input gate, candidate cell state, output gate, current cell state, and hidden layer state; $X_{t}$ is the input vector at time *t*; W is the weight coefficient matrix; and b is the bias of the corresponding cell state. The initial cell state (*C*_0_) and initial hidden state (*h*_0_) are initialized to zero vectors, which helps to avoid biasing the model towards any particular state at the beginning of the sequence.

Equation (1) uses *h_t_*_−1_ and *X_t_* with a sigmoid layer to determine data inclusion. After passing through the tanh layer using *h_t_*_−1_ and *X_t_*, data are obtained using Equation (5). Equation (3) combines long-term memory *C_t_*_−1_ and current data *C*’*_t_*. The input gate bias is *b_f_*, while the weight matrices are *W_i_*. A sigmoid layer and a dot product, together with the forget gate, enable selective data transmission. Equation (2) decides whether to forget details from a previous cell, using *W_i_* and *B_i_*. In Equations (4) and (6), *h_t_*_−1_ and *X_t_* are the inputs for the LSTM output unit, processing new data *C_t_* using the *tanh* layer to obtain the outcome.

In practice, to enhance computational efficiency, the weight matrices associated with the gates are often fused into a single matrix. This approach reduces the number of matrix multiplications required during forward propagation, thereby optimizing the computational resources and accelerating the training process. For instance, the weight matrices for the *W_i_*, *W_f_*, and *W_o_* are combined into a single weight matrix, which is then decomposed during the computation to extract the contributions for each gate.

#### 2.3.2. Improvement of LSTM Network

(1) *Convolutional Neural Network* (*CNN*)

**Figure 3:**
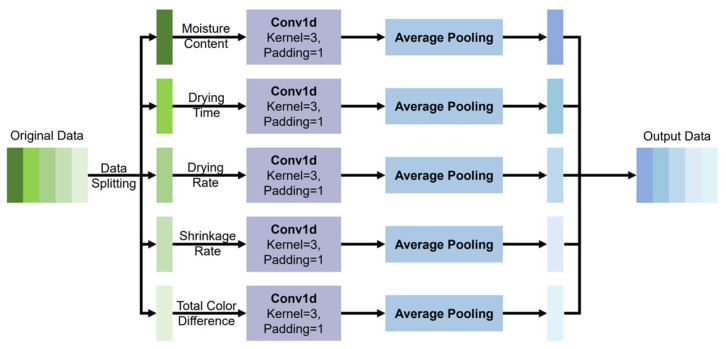
Diagram of the Convolutional Neural Network’s structure.

CNNs are capable of automatically extracting features from data by convolutional, pooling, and fully connected layers [8]. The structure of the CNN utilized in this study is depicted in Figure 3. In this study, we employ a one-dimension convolutional network to perform local feature extraction on the input data. The extracted features are then used as the input for the LSTM network, enabling the LSTM to perform sequence modeling based on more meaningful and compact representations. According to the study of Jia et al. [6], the convolutional layer utilizes kernel size = 3 and padding = 1 to capture local patterns. Furthermore, the pooling layer reduces the feature dimensions, effectively decreasing the computational complexity of the subsequent LSTM and preventing overfitting by preserving crucial features.

In conclusion, based on the advantages of CNNs mentioned above, applying a CNN to the feature extraction of LSTM can reduce the computational complexity while improving the prediction accuracy of LSTM. However, due to the fixed gating structure of LSTM, it has poor adaptability when predicting sequence data of different complexities (such as changes in the appearance quality and moisture content of the samples).

(2) *MultiHead Attention* (*MHA*)

**Figure 4:**
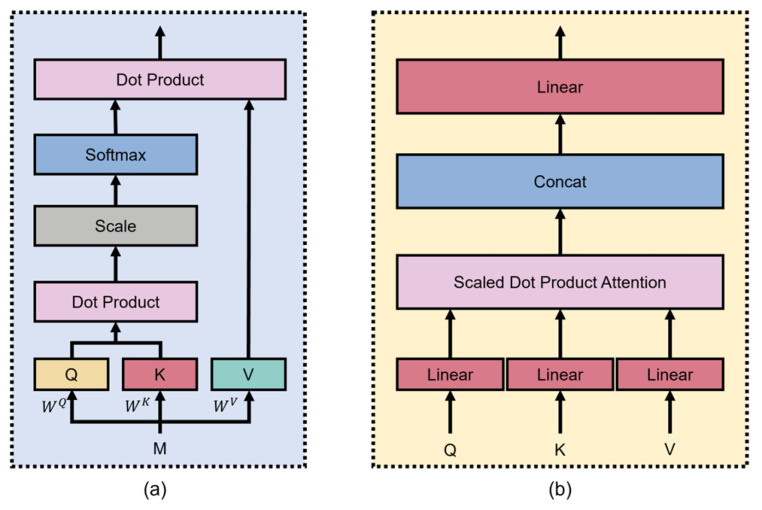
The scaled Dot-Product Attention block is shown on the left (**a**), and the MultiHead Attention block is shown on the right (**b**).

The introduction of MHA enables LSTM to focus on multiple positions in the input sequence simultaneously. This mechanism can improve the flexibility and expressiveness of the model by processing multiple attention heads in parallel (each head focuses on different features and time steps). Furthermore, MHA can also enhance the model’s ability to capture long-term dependencies and prevent the vanishing of the model gradient [11]. The attention mechanism implements scaled dot product attention through operations on three vectors: query (*Q*), key (*K*), and value (*V*). As presented in Figure 4a, during scaled dot product attention, the dot products between queries and all keys are calculated to gauge the significance of every key. Subsequently, the dot product results are divided by $\sqrt{d}$ to avoid the dot product result being too large due to the increase in dimensions, in order to avoid numerical overflow or gradient explosion, where *d* represents the dimensionality of both *K* and *Q*. After that, a SoftMax function is utilized to generate weights. These weights signify the relative importance of each key-value pair with respect to a specific query. Eventually, each attention weight is multiplied by the corresponding value to yield the output, and the relevant attention function is expressed as Equation (7) [12]:

(7) $$Attention\left(Q,K,V\right)=softmax\left(\frac{QK^{T}}{\sqrt{d}}\right)V$$

MHA can analyze the input features from various perspectives by performing parallel attention functions h times, taking different linear projections of *Q*, *K*, and *V* as inputs, as illustrated in Figure 4b. In this context, *h* represents the quantity of heads in MHA. Subsequently, the outputs of the attention mechanisms are combined and subjected to further linear projection. The formulation of MHA is given by Equations (8) and (9) [12]:

(8) $$MultiHead\left(Q,K,V\right)=concat\left(H_{1},H_{2},\ldots ,H_{h}\right)W^{O}$$

(9) $$H_{i}=Attention\left(QW_{i}^{Q},KW_{i}^{K},VW_{i}^{V}\right)$$

where ($W^{O}$, $W_{i}^{Q}$, $W_{i}^{K}$, $W_{i}^{V}$) are the linear projection matrices and *H*_*i*_ denotes the output of a single attention function. According to the study of Shu et al. [12], a value of *h* = 6 was adopted in our study.

**Figure 5:**
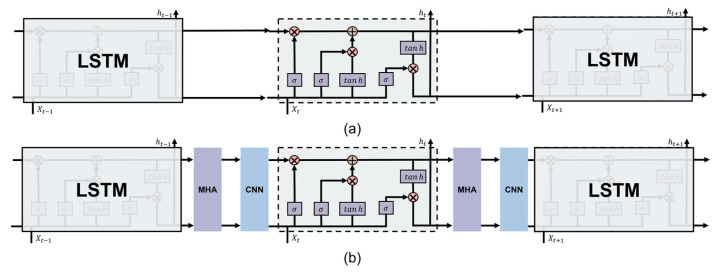
Diagram of the (**a**) LSTM and (**b**) CNN-LSTM-MHA’s structure.

The improved LSTM presented in this study is referred to as “CNN-LSTM-MHA”, and its structure is shown in Figure 5. Figure 5b shows that the MHA module and the CNN module are alternately distributed among the LSTM units. Specifically, in CNN-LSTM-MHA, after the data is preprocessed, it is input into CNN. CNN extracts the local features of the data through convolution operations and then inputs the local feature maps into LSTM to capture the temporal dependencies in the feature maps and generate hidden states. Subsequently, the hidden state is input into the MHA to calculate the correlation between time steps, highlighting the key time steps or features.

#### 2.3.3. Real-Time Control Logic of Drying Temperature Based on CNN-LSTM-MHA

**Figure 6:**
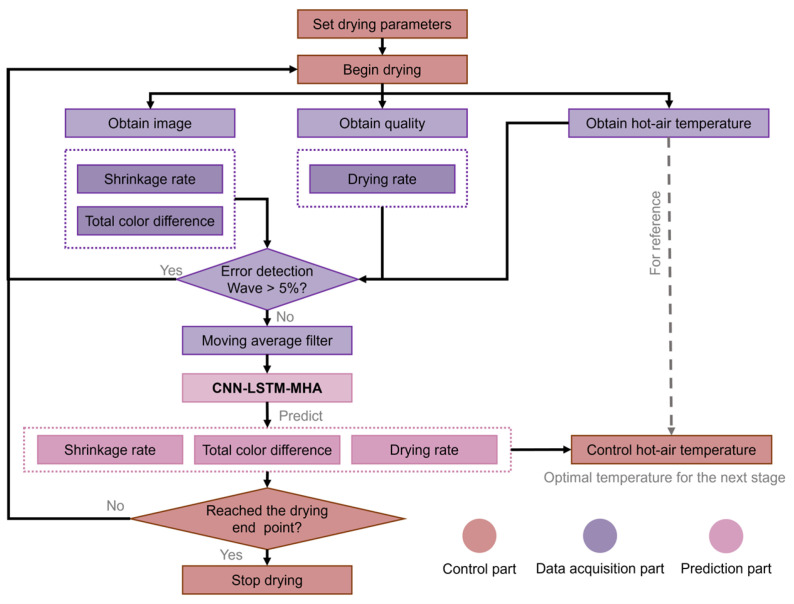
Real-time temperature control logic diagram based on CNN-LSTM-MHA (the drying endpoint refers to the moisture content of samples being less than 10% (w.b.)).

The CNN-LSTM-MHA-based real-time management approach for the HAD of yuba sticks is depicted in Figure 6. As shown, once the drying parameters (drying endpoint, initial temperature, and air velocity) are established, the system gathers the images, quality metrics, and air temperature data. Subsequently, these data are transformed into SR, Δ*E*, moisture content, and DR every 1 min and are normalized to values ranging from 0 to 1. Using these data, CNN-LSTM-MHA predicts SR, Δ*E*, and DR at different temperatures in the subsequent stages. Then, based on these predictions, the system determines the possible optimal drying temperature for the next stage and adjusts the air temperature to the best setting using a Proportion Integration Differentiation (PID) controller for the next stage. This adjustment process is based on the comprehensive scoring method [8]. Specifically, during this process, the model predicts SR, Δ*E*, and DR at different drying temperatures in the next stage. Then, the system selects the temperature setting with the highest comprehensive score of these metrics at different temperatures. Subsequently, the drying equipment is regulated to this temperature through PID. This process is carried out once every minute. Ultimately, the system assesses the current moisture content of the samples to decide if the drying process is complete. If the drying endpoint is not yet achieved, the cycle continues. Once the drying endpoint is met, the drying process terminates.

### 2.4. Model Training and Evaluation

#### 2.4.1. Model Training and Application Environment

The experimental environment of the network trained and applied in this study is shown in Table 2. The training and application environment of different models were the same.

#### 2.4.2. Model Training

The parameters were configured as follows: all input data were in ‘csv’ format, the network’s input size was set to 5 (current moisture content, drying time, DR, SR, and Δ*E*), the hidden layer size was 256, the output size was set to 3 (the next stage of DR, SR, and Δ*E*), and the mean square error (MSE) function was used to measure the loss. The Adaptive Moment Estimation (Adam) optimizer was selected, with a learning rate of 0.01 and a total of 5000 training iterations. The dataset included real-time moisture content, DR, SR, and Δ*E* normalized to the range of 0 to 1. The dataset was divided into a training set, a validation set, and a test set at a 6:2:2 ratio (time-continuous). During the model training process, the Rolling Window method (RWM) was used to extract the dataset, and the average training time was approximately 13.5 min.

#### 2.4.3. Model Evaluation

The R^2^, root mean square error (RMSE), and mean absolute error (MAE) were used as indexes to evaluate the prediction accuracy of the CNN-LSTM-MHA model, as shown in Equations (10)–(12) [13]:

(10) $$R^{2}=1-\frac{\sum_{i=1}^{n}{\left(y_{i}-{\hat{y}}_{i}\right)}^{2}}{\sum_{i=1}^{n}{\left(y_{i}-\bar{y}\right)}^{2}}$$

(11) $$RMSE=\sqrt{\frac{\sum_{i=1}^{n}{\left(y_{i}-{\hat{y}}_{i}\right)}^{2}}{n}}$$

(12) $$MAE=\frac{\sum_{i=1}^{n}\left|y_{i}-{\hat{y}}_{i}\right|}{n}$$

where $y_{i}$ is the true value; ${\hat{y}}_{i}$ is the predicted value of the CNN-LSTM-MHA network; $\bar{y}$ is the average of the true values; and n is the number of data points.

### 2.5. Drying Kinetics and Physicochemical Properties

#### 2.5.1. Drying Kinetics

The moisture ratio (MR) of yuba was calculated according to the moisture content (dry basis, d.b.), assuming a zero value of the equilibrium moisture content, as shown in Equation (13) [14]:

(13) $$MR=\frac{M_{t}}{M_{0}}$$

where $M_{0}$ is the initial moisture content of fresh yuba, the detection method of which is shown in Section 2.1, g/g, and $M_{t}$ is the moisture content of yuba when dried for *t* min, which is obtained after real-time detection of the material mass by the weighing sensor (take the average of two consecutive detections), g/g.

Also, the DR of yuba was calculated according to Equation (14) [14]:

(14) $$DR=\frac{M_{t_{1}}-M_{t_{2}}}{t_{2}-t_{1}}$$

where $M_{t1}$ and $M_{t2}$ are the moisture content (d.b.) of yuba at drying times $t_{1}$ and $t_{2}$, respectively, g/g, while $t_{1}$ and $t_{2}$ are the drying times at two weight readings, min.

#### 2.5.2. Shrinkage Rate

**Figure 7:**
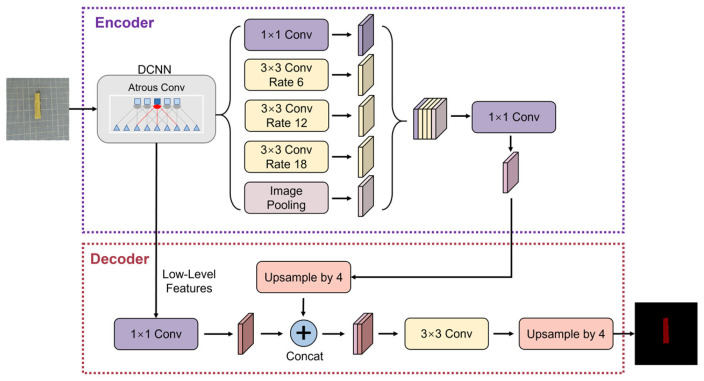
Diagram of DeepLabV3+ model.

Utilizing the DeepLabV3+ model (Figure 7) for the automatic semantic segmentation of yuba stick images during the drying process, the images of yuba were divided into a dataset at a ratio of 6:4 (quantity 2837:1892) for model training. The training parameters were set as follows: the size of the input image was 512 × 512 pixels, the batch size of the model was 8, the Adam optimizer was used as the optimizer, the maximum learning rate was 0.01, the minimum learning rate was 0.0001, the momentum was 0.9, and the number of iterations was 50. The accuracy of the trained model meets the application requirements. The mean intersection over union (MIoU), recall value, and mean average precision (mAP) were 98.38, 97.08, and 98.04, respectively. The yuba’s projected area was ascertained by tallying the segmented pixel points; subsequently, the yuba stick’s SR was computed using Equation (15) [14]:

(15) $$SR=\frac{A_{0}-A_{t}}{A_{0}}\times 100\%$$

where $A_{t}$ represents the projected area of the yuba stick when drying time is t min, pixels and $A_{0}$ is the projected area of the fresh yuba stick, pixels.

#### 2.5.3. Color

With the application of the DeepLabV3+ model for semantic segmentation, the yuba stick area was delineated, and the average values of R, G, and B color values were obtained. These values were subsequently transformed into L* (lightness/darkness), a* (redness/greenness), and b* (yellowness/blueness) values, respectively. The Δ*E* value between time intervals was then computed based on Equation (16) [15]:

(16) $$\Delta E=\sqrt{{\left(L_{0}^{*}-L_{1}^{*}\right)}^{2}+{\left(a_{0}^{*}-a_{1}^{*}\right)}^{2}+{\left(b_{0}^{*}-b_{1}^{*}\right)}^{2}}$$

Yellow is considered to be the most popular color among consumers for yuba sticks [16]. The Yellowness index (*YI*) value was then computed based on Equation (17) [15]:

(17) $$YI=\frac{142.86\times b^{*}}{L^{*}}$$

where L* and b* refer to the color parameters of dried samples.

#### 2.5.4. Rehydration Ratio

The dried samples were rehydrated following the method described by Zhou et al. [17] with minor modifications. The dried yuba sticks (5 ± 0.5 g) were first weighed, then immersed in water (1 g/20 mL of sample/water) at 85 °C for 20 min. After cooling, the remaining water on the surface was wiped away with a paper towel, and the samples were weighed after rehydration. The rehydration ratio (RR) was estimated by applying Equation (18):

(18) $$RR=\frac{W_{w}}{W_{d}}\times 100\%$$

where $W_{w}$ and $W_{d}$ refer to the sample weight after and before water absorption, respectively, g.

#### 2.5.5. Texture

The texture was analyzed following the method outlined by Sun et al. [18] with a few modifications. The texture of yuba sticks was determined by a texture profile analyzer (TA. XTPLUS/50, Stable Micro System, Surrey, UK). The yuba sticks after rehydration (refer to Section 2.5.4) were cut into 1.4 cm × 5 cm sticks, and then a P/5 cylindrical probe was used to puncture the yuba sticks at a test speed of 1 mm/s, with the trigger pressure set to 150 g, shape variable set to 70%, and probe residence time set to 1 s.

#### 2.5.6. Protein Content (PC)

The PC of yuba sticks was analyzed according to the method described by Peng et al. [19] with some modifications. The Kjeldahl method was applied to measure the total nitrogen content in the yuba sticks, and a nitrogen-to-protein conversion factor of 5.71 was used to approximate the protein content, as per the guidelines set by AOAC in 2005.

#### 2.5.7. Fat Content (FC)

The FC of yuba sticks was evaluated following the method presented by Zhu et al. [20] with slight modification. Dried yuba sticks were cracked and ground into powder. Yuba powder (5 g) was subjected to extraction in a Soxhlet siphoning-type extractor filled with petroleum ether (30~60 °C) for 6 h. Residue was dried to a constant weight in a drying oven at 45 °C for 4 h and then weighed.

#### 2.5.8. Microstructure

The cross-sectional morphology of the dried yuba sticks was inspected under a scanning electron microscope (SEM, SU3500, Hitachi, Tokyo, Japan) at a magnification of 1500×. Following a slightly adjusted method of Pei et al. [21], the samples segments underwent gold coating (Beijing Zhongquan Co., Ltd., Beijing, China) for 20 s at a pressure of 6~8 Pa prior to being scanned at a voltage of 15 kV.

### 2.6. Statistical Analysis

Data were expressed as the mean ± standard deviation (SD, *n* = 3) of three replicates. The statistical analysis was conducted using IBM SPSS statistics (version 27.0, SPSS Inc., Chicago, IL, USA). One-way analysis of variance (ANOVA) was applied to assess the statistical differences among groups, with Duncan’s multiple range test for multiple comparisons being used to identify significant differences at *p* < 0.05.

## 3. Results and Discussion

### 3.1. Evaluation Results of CNN-LSTM-MHA Model

Table 3 shows the experimental results for SR, Δ*E*, and DR compared to their predicted results from the six models. The results indicate that the CNN-LSTM-MHA model demonstrated the most precise fit between actual and predicted values, with all R^2^ values surpassing 0.9855. Furthermore, the CNN-LSTM-MHA model reflected the smallest difference between the actual and predicted values, with overall lower RMSE values (0.0001 ≤ RMSE ≤ 0.0099, 0.0001 ≤ MAE ≤ 0.0120).

**Figure 8:**
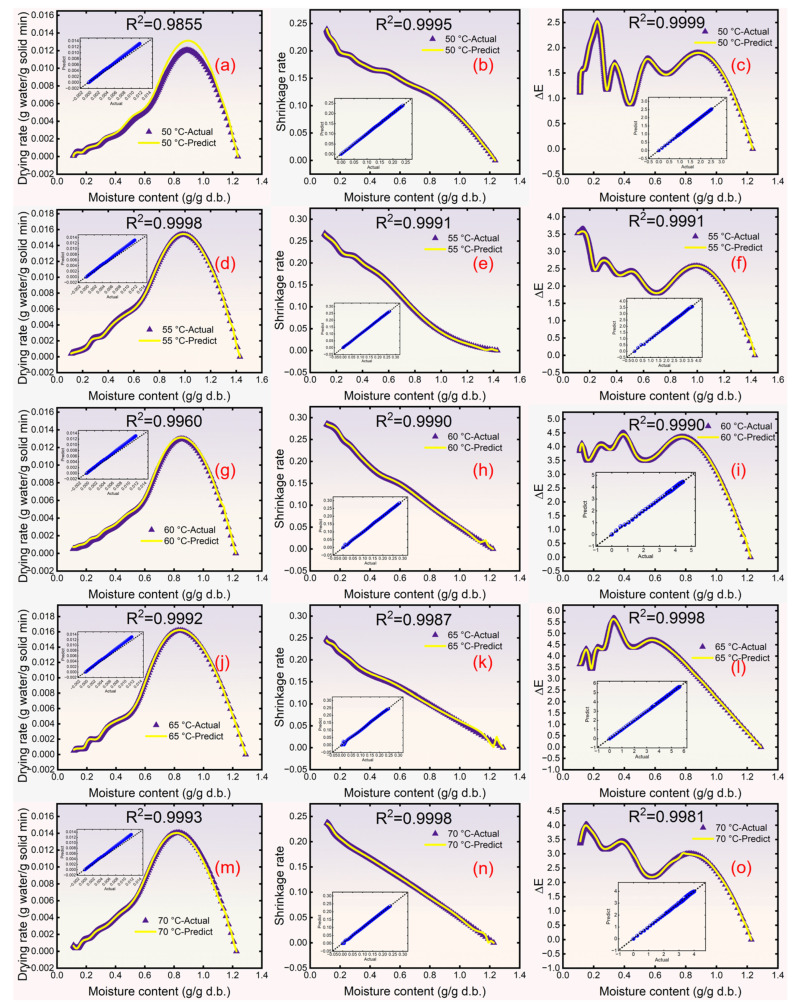
The drying rate, shrinkage rate, and total color difference prediction results of the CNN-LSTM-MHA network at 50, 55, 60, 65, and 70 °C: 50 °C-drying rate (**a**); 50 °C-shrinkage rate (**b**); 50 °C-Δ*E* (**c**); 55 °C-drying rate (**d**); 55 °C-shrinkage rate (**e**); 55 °C-Δ*E* (**f**); 60 °C-drying rate (**g**); 60 °C-shrinkage rate (**h**); 60 °C-Δ*E* (**i**); 65 °C-drying rate (**j**); 65 °C-shrinkage rate (**k**); 65 °C-Δ*E* (**l**); 70 °C-drying rate (**m**); 70 °C-shrinkage rate (**n**); 70 °C-Δ*E* (**o**).

The reason for the higher prediction accuracy of CNN-LSTM-MHA than LSTM may be divided into two aspects. Firstly, the introduction of CNN enhanced the model’s ability to extract key features from the data, especially at moments when the data fluctuates, which can provide more valuable time series data as the input to LSTM [6]. Secondly, the introduction of MHA can focus on and extract input features in parallel from different representation subspaces, such as focusing on extracting the fluctuation trend of DR on one head and focusing on extracting the correlation between DR and SR on another head. Therefore, the features extracted from these different perspectives can provide more a comprehensive input of time series data for the LSTM model, which can enable it to process the time series data better [11], as shown in Figure 8.

Compared to other models (ANN, PR, XGB, and LR), the CNN-LSTM-MHA network’s higher accuracy came from its ability to learn long-term dependencies within sequential data, thereby enabling it to capture more complex patterns and trends when dealing with variations in time series data [22]. This attribute can be explained by the unit structure of the LSTM model. Yang et al. [22] indicated that the cell states in the LSTM can transmit information across multiple time steps, which enables the LSTM to store and recall long-term information. In addition, the forget gate in the LSTM unit can selectively forget some information. These attributes enhanced the ability of LSTM to capture features in time series data with complex fluctuations and effectively inhibited the disappearance and explosion of gradients.

### 3.2. Real-Time Drying Temperature Control Results

#### 3.2.1. Real-Time Drying Temperature Control

**Figure 9:**
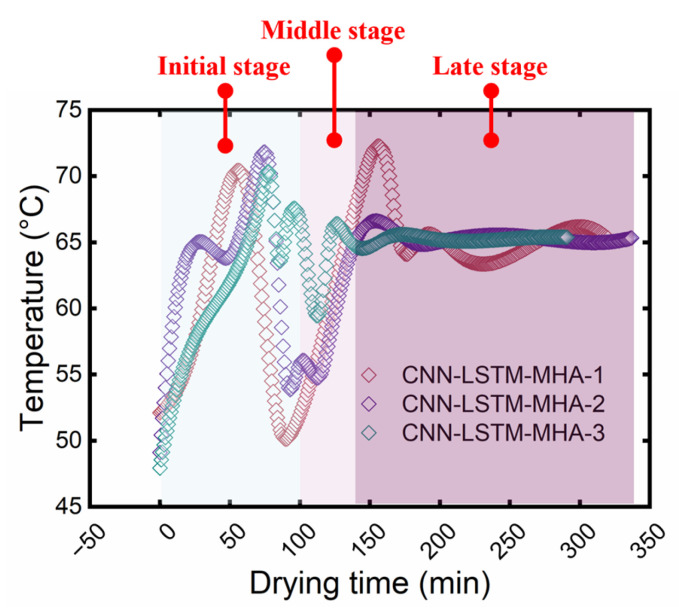
Results of real-time control of drying temperature.

The HAD temperature of yuba sticks was controlled in real time based on the CNN-LSTM-MHA network. The resulting temperature changes in the drying chamber are shown in Figure 9. Although the temperature variation trends across the three experiments were not entirely consistent, the general trend remained the same. This may be due to inherent individual variations present within the samples. This also showed the advantage of real-time drying temperature control of CNN-LSTM-MHA based on the real dynamic state of the samples, ensuring that the samples were always in the optimal drying environment. The drying temperature increased to around 70 °C during the initial drying stage (around 0~100 min). Referring to Figure 8, this temperature rise during this period may be due to the rapid increase in Δ*E*. To facilitate the rapid increase in Δ*E*, the CNN-LSTM-MHA network elevated the drying temperature to 70 °C to promote the yellowing of samples.

During the middle (around 100~130 min) and late (around 130~330 min) stages, the drying temperature decreased rapidly and then increased to approximately 65 °C. CNN-LSTM-MHA decreased the temperature rapidly at this stage with the likely intention of inhibiting the shrinkage of the samples when the moisture content ranged from 0.6 to 0.4 g/g (d.b.). This was confirmed in the study of Jiang et al. [23], whose research on carrot slices also indicated that a decrease in drying temperature inhibited SR, which is consistent with the general understanding. In the late drying stage, as the moisture was removed, the partial pressure of water vapor between the samples and the drying medium gradually decreased, leading to a decrease in DR. Therefore, to speed up dehydration, it was necessary to use high temperatures to provide a greater driving force for mass transfer [24].

#### 3.2.2. Accuracy of CNN-LSTM-MHA Network Real-Time Prediction

**Figure 10:**
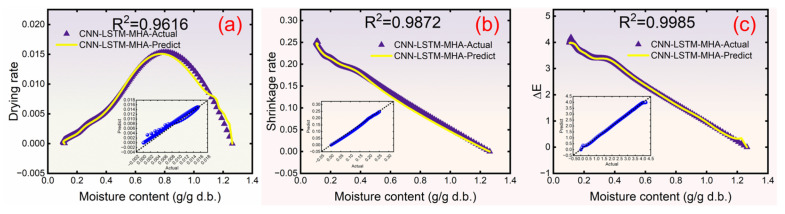
The drying rate (**a**), shrinkage rate (**b**), and total color difference (**c**) real-time prediction results of the CNN-LSTM-MHA network.

Figure 10 shows the changes in the observed and predicted values of the DR, SR, and Δ*E* of yuba sticks. The point-by-point predictions in Figure 10 rely on the model’s sequential learning ability, which integrates historical time series data to capture dynamic correlations between drying parameters. This ensures prediction robustness even with frequent temperature adjustments. Figure 10a shows a slight deviation between actual and predicted values, with an R^2^ value of 0.9616, indicating a negative effect on the prediction of DR under frequent variations in drying temperature. This may be due to the limited amount of data available for the network’s reference during early drying stages, which compromised its predicted accuracy for time series data variations, such as DR. However, compared with our previous research [8], the prediction accuracy of the network was significantly improved (R^2^ increased from 0.8475 to 0.9410–0.9998). As shown in Figure 10b,c, the R^2^ values between the predicted and actual values of the CNN-LSTM-MHA were 0.9872 and 0.9985, respectively, indicating accurate predictions of the yuba sticks’ SR and Δ*E* changes after altering the drying temperature. Consequently, the data variation curve predicted by the CNN-LSTM-MHA network provides a basis for controlling drying temperatures.

### 3.3. Drying Kinetics and Physicochemical Properties

#### 3.3.1. Drying Kinetics

**Figure 11:**
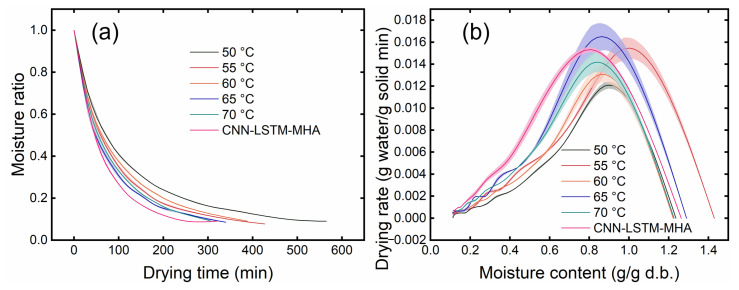
Moisture ratio (**a**) and drying rate curves (**b**) of yuba sticks under different drying temperature control methods.

The MR and DR curves of yuba sticks under different drying temperature control methods are shown in Figure 11. According to Figure 11a, increasing the drying temperature reduces the drying time. The time required to dry the CNN-LSTM-MHA samples until the final moisture content (10%) was reached was 320.59 min. This achieved a 43.36%, 25.06%, 17.53%, and 5.88% reduction in drying time compared with the 50, 55, 60, and 65 °C drying scenarios, respectively. However, the drying time for the CNN-LSTM-MHA-treated samples increased by 4.92% compared to the samples dried at 70 °C.

There is a positive correlation between drying temperature and heat and mass transfer. The increase in drying temperature improves the heat and mass transfer efficiency, thereby shortening the drying time [24]. This is the typical drying behavior of many food samples [25]. This also clarifies why the CNN-LSTM-MHA-treated samples’ drying time was longer than 70 °C, where the drying temperature was maintained between 65 and 70 °C during the drying process.

As presented in Figure 11b, a rapid increase in DR was observed, followed by a gradual decrease during the initial drying stage. The initial increase in DR can be attributed to the excess moisture on the surface of the yuba sticks, leading to a rapid moisture removal rate. As drying advanced, surface moisture evaporation became less significant, while inherent moisture diffusion within the samples started to dominate the process [26]. These observations align with studies on persimmon, blueberries, and kiwifruit slices [27,28,29].

It must be mentioned that a higher DR was observed in the late drying stage for the CNN-LSTM-MHA-treated samples than others. This higher DR can be attributed to the pulsed temperature changes (50–70 °C) during the initial and middle stages, avoiding the damage to the microstructure caused by thermal stress that occurs under continuous high temperatures. Wang et al. [30] also pointed out that less damage to the microstructure during drying could promote the migration and diffusion of moisture. In addition, higher drying temperatures in the late drying stage further enhanced heat and mass transfer during the drying process. This effectively shortened the drying time required for the CNN-LSTM-MHA process compared to other constant-temperature drying scenarios [24].

#### 3.3.2. Shrinkage Rate

**Figure 12:**
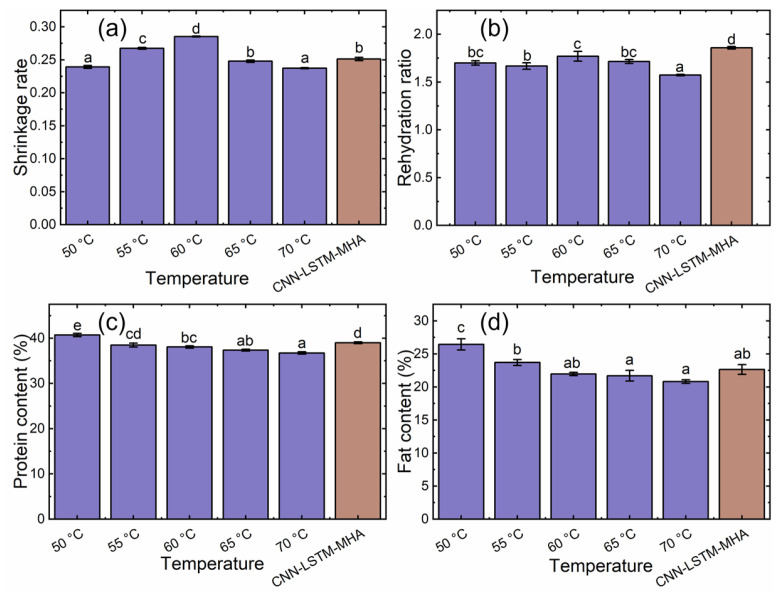
Shrinkage rate (**a**); rehydration ratio (**b**); protein content (**c**); and fat content (**d**) under different drying temperature control methods. The different lowercase letter indicates a statistically significant difference at *p* < 0.05.

The SRs of the yuba sticks under different drying temperature control methods are summarized in Figure 12a. As the drying temperature increased from 50 to 60 °C, the SRs of the samples gradually increased from 11.74% to 19.26%, which was consistent with expectations. However, at 65 and 70 °C, the SR decreased by 13.14% and 16.82%, respectively, which is unexpected, as higher temperatures typically increase shrinkage. The SR of CNN-LSTM-MHA-treated samples was significantly lower by 6.02% and 11.95% compared to 55 and 60 °C, but slightly higher by 5.01%, 1.37%, and 5.86% compared to 50, 65, and 70 °C, respectively.

**Figure 13:**
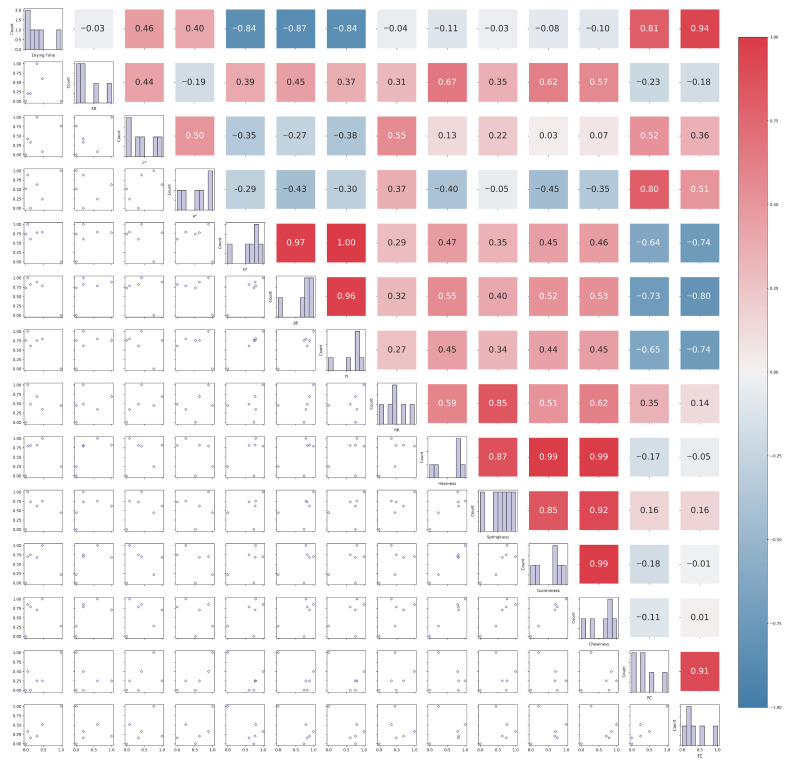
Correlation analysis among drying kinetics and physicochemical properties under different control methods of drying temperature (SR: Shrinkage rate; Δ*E*: total color difference; *YI*: Yellowness index; RR: rehydration ratio; PC: protein content; FC: fat content).

Li et al. [31] reported that moisture evaporation during drying is usually accompanied by significant volume shrinkage, which is mostly affected by moisture migration and temperature. The increase in SR between 50 and 60 °C can be attributed to the diminishment of viscoelastic stresses within the pores due to the loss of moisture, leading to a subsequent decrease in the inherent pore pressures [32]. This is supported by Figure 13, which shows a weak positive correlation between SR and hardness (*r* = 0.67), indicating that the cause of shrinkage was related to the reduction or disappearance of micropores in the samples, as this could increase the hardness of the samples, as is widely observed [31]. However, the decrease in SR between 60 and 70 °C may be associated with the reduction in drying time, which helped to maintain the microstructure [33]. When the temperature was above 60 °C, the positive impact of the shortened drying time predominated. This was also confirmed in the study of Li et al. [31], which pointed out that the shrinkage behavior of materials in the drying process is a synergistic effect of the drying method, drying temperature, and drying time, where the shortening of drying time can inhibit shrinkage. Additionally, this phenomenon has also been observed in studies on potatoes by McMinn & Magee [34] and Wang & Brennan [35]. They believed that high temperature reduced the SR because of the shell-hardening effect of the samples at high temperatures, restricting their deformation and inhibiting their shrinkage.

The CNN-LSTM-MHA-controlled samples did not exhibit the minimum SR, possibly because this network focused more on the automatic optimization of DR and color, which could result in the drying temperature at some stages not being the most suitable for optimizing SR. This is supported by Figure 8 and Figure 9. For example, before 50 min, the drying temperature fluctuated between 55 and 65 °C, while this temperature range increased the SR of the samples, as shown in Figure 12a. However, this temperature range had a favorable effect on yuba sticks’ DR, as explained in Section 3.3.1. This indicated that the selection of the optimal drying temperature by the CNN-LSTM-MHA network was based on the balance between the DR, SR, and color of yuba sticks, which is beneficial for comprehensively improving the drying performance of samples.

#### 3.3.3. Color

Color is a key quality indicator for dried yuba sticks and significantly impacts consumer acceptance [16]. The effects of different drying temperature control methods on the color parameters of yuba sticks are presented in Table 4. There were no significant differences in the samples’ L* and a* values (*p* > 0.05), indicating that the drying temperature did not affect the samples’ brightness/darkness and redness/greenness values. The lowest *YI* value was obtained at 50 °C, significantly lower than that of the high-temperature-dried samples (*p* < 0.05). The b* and Δ*E* of the samples were consistent with the changing trend of *YI*, indicating that the color change during drying was primarily yellow.

The yellowing of high-temperature-dried samples can be ascribed to non-enzymatic browning (Millard reaction) during drying, resulting in a more yellowish color [36]. Higher b* values were associated with higher *YI* values in high-temperature-dried samples, providing a strong correlation (0.96 ≤ *r* ≤ 1) between b*, Δ*E*, and *YI*, as demonstrated in Figure 13.

The CNN-LSTM-MHA-treated samples exhibited the highest *YI* values, signifying a more pronounced yellow color. This greater *YI* is possibly due to the real-time drying temperature control promoting the Maillard reaction. In their review, Pathare et al. [15] pointed out that temperature reduction would inhibit the occurrence of the Maillard reaction. In conclusion, the CNN-LSTM-MHA-based real-time temperature control technology can represent a recommended scenario for yuba stick drying, achieving an impressive final product with an excellent color.

#### 3.3.4. Rehydration Ratio

The capacity of a dried substance to revert to its initial condition upon rehydration reflects its structural integrity, which can be assessed through the rehydration process [14]. The RR results of the dried yuba sticks are shown in Figure 12b. Samples subjected to HAD at between 50 and 65 °C displayed no significant differences in RR. This indicated that a low drying temperature had a limited effect on the samples’ rehydration performance. However, the RR significantly reduced to the lowest level after samples were subjected to the 70 °C drying conditions, showing the worst water absorption capacity. Notably, the CNN-LSTM-MHA-managed samples exhibited the highest RR (*p* < 0.05), being 9.33%, 11.48%, 5.06%, 8.40%, and 18.15% higher than the constant-temperature treatments from 50 to 70 °C, respectively, suggesting superior water absorption capabilities.

The RR of samples dried at 70 °C decreased, in accordance with Aradwad et al.’s [37] observation that the RR is closely related to the porosity and cavity of the sample microstructure. The samples dried at 70 °C precipitated a large amount of oil and solidified into a shell on their surface, which made it difficult for external water to be transported through the micropores in the samples. The highest RR of CNN-LSTM-MHA-treated samples can be attributed to the CNN-LSTM-MHA network’s dynamic adjustment of temperatures during the drying process, which optimized the DR and facilitated uniform moisture evaporation. This control mitigated inherent stress and damage within the samples, suppressed pore tearing, and maintained a more intact structure of the protein–fat network (SPFN), consistent with Ogawa & Adachi’s review [38] of pasta’s RR.

#### 3.3.5. Texture

Texture is a physical characteristic determined by the composition and microstructure [18]. The hardness, springiness, cohesiveness, gumminess, and chewiness of rehydrated samples after drying under different drying temperature control methods are summarized in Table 5. The hardness of samples dried at 50 and 70 °C was significantly lower than that of the samples dried at other temperatures (*p* < 0.05), exhibiting an initial increase followed by a decrease with increasing temperature. Springiness showed a similar trend, increasing up to 60 °C and then decreasing, but without significant differences (*p* > 0.05), indicating that the drying temperature had little impact on the yuba sticks’ springiness. The other textural properties of the samples, particularly gumminess, exhibited a similar trend to hardness, resulting in more palatable yuba with reduced gumminess at 50 and 70 °C. The samples dried at 55 °C exhibited significantly higher chewiness compared to the other samples (*p* < 0.05).

It is generally believed that the hardness of samples increases with higher drying temperatures due to less damage occurring to the microstructure at lower temperatures, resulting in a softer texture upon rehydration. In this study, the decrease in hardness at high temperatures may be attributed to the SR of the high-temperature-treated samples being low and the inherent structure being loose (*r* = 0.67). The 50 °C samples had lower gumminess, which may be due to the rate of protein cross-linking to form a film being slower at lower drying temperatures, potentially leading to a looser structure and decreased gumminess, aligning with the findings of Gennadios & Weller [39]. The reduced gumminess at 70 °C may be attributed to the minimum RR, where the samples absorbed the least amount of water, thus diminishing the gumminess of the samples. This was demonstrated in a study of sweet potato starch noodles by Xiang et al. [40], in which the gumminess of noodles decreased with decreasing RR. The LSTM-treated samples exhibited higher hardness and chewiness, as well as the highest springiness, which may be more suitable for consumers who prefer a crisp texture in yuba sticks.

#### 3.3.6. Protein Content

The PC under different drying temperature control methods is presented in Figure 12c. As the temperature increased, the PC gradually decreased. The PC of the 50 °C samples was significantly higher than that of the samples treated at 55 to 70 °C and those treated with LSTM (*p* < 0.05) by 5.86%, 6.99%, 8.96% and 10.85%, respectively, indicating that low-temperature drying was beneficial to the retention of protein in yuba sticks. The PC of the LSTM-treated samples was not significantly different from those of the samples treated at 55 °C (*p* > 0.05), but was significantly higher than those of the samples dried at 60 to 70 °C (*p* < 0.05), by 2.46%, 4.35%, and 6.15%, respectively, suggesting that the temperature control method based on the LSTM can shorten the drying time to a certain degree and improve the retention rate of proteins.

The reason for the decrease in PC with increasing temperature may be that high temperature promoted protein hydrolysis into polypeptides and amino acids and participated in the Maillard reaction to produce ketones, aldehydes, and other volatile compounds. This is consistent with Liao et al.’s [41] study on the steaming and drying of black soybeans, which also attributed the decrease in PC to the intense Maillard reaction involving sugar and protein in the samples under a high-temperature environment, which is commonly considered to constitute a risk of nutrient loss for soybean products. This is further confirmed in Figure 13, where PC was negatively correlated with b*, Δ*E,* and *YI* (−0.73 ≤ *r* ≤ −0.64). In addition, dos Santos et al. [42] reported that not only was the PC of soybeans after convection drying related to drying temperature, but also the denaturation and degradation rates of proteins were inhibited with a shorter drying time, which effectively explains why the PC of LSTM-treated samples was significantly higher than that of samples treated at 60–70 °C (*p* < 0.05).

#### 3.3.7. Fat Content

The FC under different drying temperature control methods is shown in Figure 12d. As shown in Figure 12d, the FC of the samples decreased by 10.33%, 16.94%, 17.91%, and 21.25%, respectively, as the drying temperature increased from 50 to 70 °C. In addition, LSTM-treated samples maintained a higher FC than samples treated at 60 to 70 °C (3.18%, 4.39%, and 8.83%, respectively), except for those dried at 50 and 55 °C, indicating that LSTM-treated samples exhibited a more desirable fat retention rate.

Liao et al. [41] reported that during the heating process, especially at high temperatures, lipids undergo thermal degradation and secondary reactions of thermal degradation products, thus further producing volatile compounds. This phenomenon may have contributed to the decrease in fat content to some extent. Further research is needed to understand the underlying mechanisms involved in the variation in FC during drying. On the other hand, in the study by Farmer [43], it was argued that lipid oxidation and Maillard reactions usually do not occur alone, and that each reaction may be modified by the reactants, intermediates, and products of others. Many of the effects of lipid–Maillard interactions appear to be due to reactions between carbonyl compounds (from the degradation of lipids or sugars) and amines (NH_3_, amino acids, ethanolamine) or mercaptans (H_2_S, mercaptoacetaldehyde). This is demonstrated by the results in Figure 13, which show that FC is negatively correlated with b*, Δ*E,* and *YI* (−0.74 ≤ *r* ≤ −0.80). LSTM-treated samples were exposed to low temperatures for some time during the drying process, which may have reduced the intensity of the above reaction, therefore leading to a higher FC than in samples that are dried at high temperatures throughout the process.

#### 3.3.8. Microstructure

**Figure 14:**
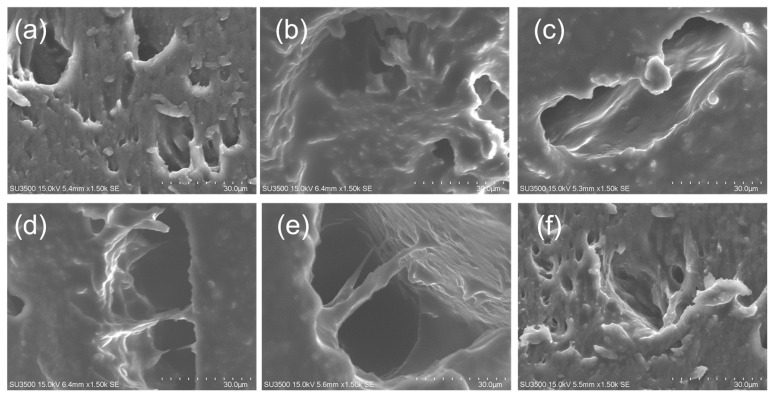
Effects of different drying temperature control methods on the microstructures of the cross-section of yuba: (**a**–**f**) yuba microstructures dried at 50, 55, 60, 65, and 70 °C and with CNN-LSTM-MHA-based temperature real-time control.

The microstructures of the yuba stick cross-sections after dehydration at different drying temperatures were observed by means of SEM and are shown in Figure 14. The dried samples treated at 50 and 55 °C had smaller pores than those dried at higher temperatures, and the SPFN was more complete. A significant increase in pore size was observed upon surpassing a drying temperature of 60 °C, accompanied by the tearing of pores. Dense pores were observed in the CNN-LSTM-MHA-treated samples without the pores being torn. This preservation of the SPFN’s integrity, relative to other temperature drying methods, likely contributes to a superior rehydration capacity.

The main reason for the small pore size of low-temperature-treated samples is that the moisture evaporated more slowly at lower temperatures and had less impact on inherent structures, consistent with the findings of Aksoy et al. [44]. This phenomenon can be elucidated by the significant acceleration in the moisture evaporation rate as the temperature increases, reducing the drying time. The SPFN undergoes short-term intense stress, precipitating the emergence of large and torn pores [45].

The microstructure of CNN-LSTM-MHA-treated samples was well maintained, which may be attributed to the slow and gradual temperature increase in the initial drying stage. This avoided accelerated moisture evaporation caused by rapid temperature increases and effectively reduced microstructural damage. This finding is similar to the conclusion of Yao et al. [46] on the microstructural changes of fruits and vegetables during drying. In the middle drying stage, the CNN-LSTM-MHA network reduced the temperature, which also might reduce the migration rate of moisture, contributing to the microstructure maintenance [46]. During drying, the moisture is gradually removed, and the partial pressure of water vapor between the samples and the drying medium gradually decreases, resulting in a decrease in the DR, as shown in Figure 11b. Therefore, in the late drying stage, even if the temperature was raised to about 65 °C, the moisture evaporation rate would still be low and would not damage the microstructure.

## 4. Conclusions

In this study, a CNN and MHA were introduced to reduce the prediction accuracy of the LSTM network when drying parameters change frequently. The CNN-LSTM-MHA network was employed to predict the real-time DR, SR, and Δ*E* changes in samples during drying, aiming to optimize drying temperature in real time. Compared with other commonly used networks, the CNN-LSTM-MHA network demonstrated the highest accuracy (R^2^: 0.9855–0.9999; RMSE: 0.0001–0.0099; MAE: 0.0001–0.0120). Even when the drying temperature changed frequently, the prediction accuracy of CNN-LSTM-MHA for DR, SR, and Δ*E* was still high (R^2^ > 0.9616). In addition, based on the CNN-LSTM-MHA network, an intelligent real-time temperature control system was developed and compared to constant-temperature scenarios (50, 55, 60, 65, and 70 °C), showing significant improvements in drying time (5.88–43.36%), SR (6.02–11.95%), color, RR (5.06–18.15%), texture, PC (2.46–6.15%), FC (3.18–8.83%), and microstructure. These results demonstrate the potential application of this novel drying strategy in dried yuba stick processing. However, the limitation of this study lies in the fact that only a single drying parameter (temperature) was controlled, which inhibited the overall improvement of the drying process. In future studies, multiple parameters for controlling the drying process, such as humidity and air velocity, should also be considered to comprehensively optimize the drying process.

## Figures and Tables

**Table 1:** Experimental design.

Experimental Number	Hot-Air Temperature Control Mode	Air Velocity (m/s)
1	Hot-air temperature is kept at 50 °C	1.5
2	Hot-air temperature is kept at 55 °C	
3	Hot-air temperature is kept at 60 °C	
4	Hot-air temperature is kept at 65 °C	
5	Hot-air temperature is kept at 70 °C	
CNN-LSTM-MHA	Intelligent control of hot-air temperature based on samples’ drying rate and appearance quality	

**Table 2:** Experimental configuration.

Environment	Configuration	Parameter
Training environment	CPU	Intel Core i7-13700K
	GPU	NVIDIA GeForce RTX 4060Ti 16 GB
	Operating system	Windows 11
	Accelerated environment	CUDA 12.2
	Models frame	PyTorch 2.0.1, torchvision 0.15.2, torchaudio 2.0.2
Application environment	Device	Raspberry PI 4B
	CPU	BCM2711
	Operating system	Raspberry PI OS 3.3
	Models frame	PyTorch 2.0.1, torchvision 0.15.2, torchaudio 2.0.2

**Table 3:** Comparing and analyzing the comprehensive performance of CNN-LSTM-MHA model based on the parameters such as R^2^, RMSE, and MAE.

Models	Temperature (°C)	Metric	R^2^	RMSE	MAE	Models	Temperature (°C)	Metric	R^2^	RMSE	MAE
LR	50	SR	0.9553	0.0091	0.0080	PR	50	SR	0.9992	0.0012	0.0007
		Δ*E*	0.0873	0.4025	0.3332			Δ*E*	0.9883	0.0456	0.0343
		DR	0.7731	0.0014	0.0006			DR	0.9927	0.0002	0.0001
	55	SR	0.9900	0.0070	0.0049		55	SR	0.9997	0.0011	0.0007
		Δ*E*	0.6649	0.3851	0.3464			Δ*E*	0.9935	0.0535	0.0340
		DR	0.7383	0.0021	0.0009			DR	0.9920	0.0004	0.0001
	60	SR	0.9875	0.0080	0.0068		60	SR	0.9864	0.0083	0.0081
		Δ*E*	0.1816	0.4897	0.3209			Δ*E*	0.9853	0.0656	0.0398
		DR	0.7204	0.0018	0.0008			DR	0.9909	0.0003	0.0001
	65	SR	0.9878	0.0063	0.0055		65	SR	0.9995	0.0013	0.0008
		Δ*E*	0.2455	0.7755	0.6286			Δ*E*	0.9941	0.0688	0.0549
		DR	0.6779	0.0025	0.0012			DR	0.9901	0.0004	0.0002
	70	SR	0.9939	0.0043	0.0036		70	SR	0.9995	0.0012	0.0009
		Δ*E*	0.7550	0.3377	0.2635			Δ*E*	0.9903	0.0673	0.0450
		DR	0.6733	0.0023	0.0011			DR	0.9899	0.0004	0.0002
XGB	50	SR	0.8486	0.0678	0.0492	ANN	50	SR	0.9782	0.0064	0.0034
		Δ*E*	0.8503	0.0630	0.0483			Δ*E*	0.9898	0.0064	0.0024
		DR	0.8526	0.0924	0.0641			DR	0.9957	0.0002	0.0001
	55	SR	0.8532	0.0993	0.0742		55	SR	0.9984	0.0018	0.0011
		Δ*E*	0.8496	0.0683	0.0579			Δ*E*	0.9981	0.0287	0.0183
		DR	0.8525	0.1013	0.0752			DR	0.9934	0.0003	0.0001
	60	SR	0.8520	0.0942	0.0762		60	SR	0.9979	0.0004	0.0003
		Δ*E*	0.8201	0.0448	0.0257			Δ*E*	0.9975	0.0270	0.0205
		DR	0.8519	0.1011	0.0746			DR	0.9958	0.0002	0.0001
	65	SR	0.8483	0.0869	0.0680		65	SR	0.9989	0.0004	0.0004
		Δ*E*	0.8363	0.0602	0.0383			Δ*E*	0.9997	0.0156	0.0126
		DR	0.8518	0.1057	0.0785			DR	0.9943	0.0003	0.0002
	70	SR	0.8475	0.0886	0.0679		70	SR	0.9975	0.0028	0.0015
		Δ*E*	0.8385	0.0636	0.0463			Δ*E*	0.9989	0.0075	0.0030
		DR	0.8517	0.1087	0.0840			DR	0.9954	0.0003	0.0001
LSTM	50	SR	0.9935	0.0082	0.0044	CNN-LSTM-MHA	50	SR	0.9995	0.0010	0.0009
		Δ*E*	0.9957	0.0275	0.0197			Δ*E*	0.9999	0.0040	0.0027
		DR	0.9922	0.0003	0.0002			DR	0.9855	0.0003	0.0002
	55	SR	0.9999	0.0007	0.0004		55	SR	0.9991	0.0006	0.0005
		Δ*E*	0.9982	0.0281	0.0192			Δ*E*	0.9991	0.0099	0.0024
		DR	0.9903	0.0006	0.0004			DR	0.9998	0.0001	0.0000
	60	SR	0.9961	0.0045	0.0025		60	SR	0.9990	0.0009	0.0006
		Δ*E*	0.9991	0.0162	0.0123			Δ*E*	0.9990	0.0172	0.0107
		DR	0.9989	0.0008	0.0005			DR	0.9960	0.0002	0.0002
	65	SR	0.9989	0.0005	0.0005		65	SR	0.9987	0.0021	0.0015
		Δ*E*	0.9969	0.0495	0.0413			Δ*E*	0.9998	0.0110	0.0084
		DR	0.9982	0.0005	0.0004			DR	0.9992	0.0001	0.0001
	70	SR	0.9989	0.0005	0.0004		70	SR	0.9998	0.0007	0.0003
		Δ*E*	0.9947	0.0495	0.0390			Δ*E*	0.9981	0.0091	0.0120
		DR	0.9990	0.0004	0.0003			DR	0.9993	0.0001	0.0001

Notes: LR: linear regression; PR: polynomial regression; XGB: XGBoost; ANN: Artificial Neural Network; LSTM: Long Short-Term Memory; CNN-LSTM-MHA: integrating a Convolutional Neural Network and MultiHead Attention with Long Short-Term Memory.

**Table 4:** The L*, a*, b*, Δ*E*, and *YI* of samples.

Experimental Number	L*	a*	b*	Δ*E*	*YI*
1	80.39 ± 1.82 ^a^	−2.58 ± 0.47 ^a^	3.70 ± 0.48 ^a^	1.11 ± 0.11 ^a^	6.57 ± 0.79 ^a^
2	79.54 ± 1.22 ^a^	−3.05 ± 0.42 ^a^	5.74 ± 0.10 ^b^	3.53 ± 0.07 ^b^	10.28 ± 0.21 ^b^
3	80.68 ± 1.09 ^a^	−2.81 ± 0.37 ^a^	5.70 ± 0.64 ^b^	3.84 ± 0.32 ^b^	10.09 ± 0.64 ^b^
4	79.85 ± 1.08 ^a^	−3.20 ± 0.59 ^a^	5.26 ± 0.49 ^b^	3.64 ± 0.55 ^b^	9.41 ± 0.88 ^b^
5	79.44 ± 1.49 ^a^	−2.88 ± 0.14 ^a^	5.61 ± 0.99 ^b^	3.36 ± 0.44 ^b^	10.09 ± 0.84 ^b^
CNN-LSTM-MHA	79.96 ± 1.13 ^a^	−2.65 ± 0.26 ^a^	6.28 ± 0.33 ^b^	4.19 ± 0.28 ^b^	11.22 ± 0.56 ^b^

Note: The different lowercase letter indicates a statistically significant difference at *p* < 0.05.

**Table 5:** The hardness, springiness, gumminess, and chewiness of samples.

Temperature (°C)	Hardness (N)	Springiness	Gumminess	Chewiness
50	199.70 ± 1.48 ^a^	2.26 ± 0.10 ^a^	14,483.46 ± 1616.28 ^a^	32,690.11 ± 4093.73 ^ab^
55	242.86 ± 0.37 ^b^	2.40 ± 0.14 ^a^	20,001.36 ± 1153.09 ^b^	48,001.75 ± 5603.90 ^c^
60	232.55 ± 1.00 ^b^	2.34 ± 0.06 ^a^	17,748.63 ± 922.44 ^b^	41,833.09 ± 1488.59 ^bc^
65	232.10 ± 0.46 ^b^	2.39 ± 0.20 ^a^	18,234.54 ± 245.45 ^b^	43,517.88 ± 3129.51 ^bc^
70	185.45 ± 0.82 ^a^	2.06 ± 0.53 ^a^	12,920.11 ± 795.87 ^a^	26,846.57 ± 8285.24 ^a^
LSTM	230.99 ± 0.92 ^b^	2.51 ± 0.14 ^a^	17,883.04 ± 834.14 ^b^	44,903.90 ± 3374.35 ^c^

Note: Different lowercase letters indicate statistically significant difference at *p* < 0.05.

## Data Availability

The original contributions presented in this study are included in the article. Further inquiries can be directed to the corresponding author.

## References

[1] Jimoh K.A., Hashim N., Shamsudin R., Che Man H., Jahari M. (2023). Recent Advances of Optical Imaging in the Drying Process of Grains—A Review. J. Stored Prod. Res..

[2] Mahmood N., Liu Y., Munir Z., Zhang Y., Niazi B.M.K. (2022). Effects of Hot Air Assisted Radio Frequency Drying on Heating Uniformity, Drying Characteristics and Quality of Paddy. LWT.

[3] Usama M., Ali Z., Ndukwu M.C., Sathyamurthy R. (2023). The Energy, Emissions, and Drying Kinetics of Three-Stage Solar, Microwave and Desiccant Absorption Drying of Potato Slices. Renew. Energy.

[4] Vilas C., Alonso A.A., Balsa-Canto E., López-Quiroga E., Trelea I.C. (2020). Model-Based Real Time Operation of the Freeze-Drying Process. Processes.

[5] Nadian M.H., Abbaspour-Fard M.H., Martynenko A., Golzarian M.R. (2017). An Intelligent Integrated Control of Hybrid Hot Air-Infrared Dryer Based on Fuzzy Logic and Computer Vision System. Comput. Electron. Agric..

[6] Jia Z., Liu Y., Xiao H. (2024). Deep Learning Prediction of Moisture and Color Kinetics of Apple Slices by Long Short-Term Memory as Affected by Blanching and Hot-Air Drying Conditions. Processes.

[7] Dong F., Bi Y., Hao J., Liu S., Yi W., Yu W., Lv Y., Cui J., Li H., Xian J. (2024). A New Comprehensive Quantitative Index for the Assessment of Essential Amino Acid Quality in Beef Using Vis-NIR Hyperspectral Imaging Combined with LSTM. Food Chem..

[8] Guo J., Liu Y., Lei D., Peng Z., Mowafy S., Li X., Jia Z., Ai Z., Xiao H. (2025). Combining DeepLabV3 + and LSTM for Intelligent Drying Strategy Optimization in Fruits and Vegetables Based on Appearance Quality: A Case Study of *Pleurotus eryngii*. Comput. Electron. Agric..

[9] Su J.-F., Huang Z., Yuan X.-Y., Wang X.-Y., Li M. (2010). Structure and Properties of Carboxymethyl Cellulose/Soy Protein Isolate Blend Edible Films Crosslinked by Maillard Reactions. Carbohydr. Polym..

[10] Meng J., Li C., Tao J., Li Y., Tong Y., Wang Y., Zhang L., Dong Y., Du J. (2023). RNN-LSTM-Based Model Predictive Control for a Corn-to-Sugar Process. Processes.

[11] Deng Z., Hou Y., Jolfaei A., Zhou W., Farivar F., Haghighi M.S. (2024). Multihead Attention-Based Multiscale Graph Convolution Network for ITS Traffic Forecasting. IEEE Syst. J..

[12] Shu Y., Kang J., Zhou M., Yang Q., Zeng L., Yang X. (2023). Load Disaggregation Based on a Bidirectional Dilated Residual Network with Multihead Attention. Electronics.

[13] Wengang H., Xiyu W., Jiajie M., Ping G., Lei W. (2024). Operation Prediction of Open Sun Drying Based on Mathematical-Physical Model, Drying Kinetics and Machine Learning. Innov. Food Sci. Emerg. Technol..

[14] Mowafy S., Guo J., Lei D., Liu Y. (2024). Application of Novel Blanching and Drying Technologies Improves the Potato Drying Kinetics and Maintains Its Physicochemical Attributes and Flour Functional Properties. Innov. Food Sci. Emerg. Technol..

[15] Pathare P.B., Opara U.L., Al-Said F.A.-J. (2013). Colour Measurement and Analysis in Fresh and Processed Foods: A Review. Food Bioprocess Technol..

[16] Tao Y., Shen Y., Xu L., Tang Q., Yang H. (2023). Design and Experimental Research of Intelligent Inspection and Classification System for Yuba Skin Quality. Appl. Sci..

[17] Zhou Y.-H., Pei Y.-P., Sutar P.P., Liu D.-H., Deng L.-Z., Duan X., Liu Z.-L., Xiao H.-W. (2022). Pulsed Vacuum Drying of Banana: Effects of Ripeness on Drying Kinetics and Physicochemical Properties and Related Mechanism. LWT.

[18] Sun W., Li M., Zhang Y., Ai Z., Lei D., Pei Y., Liu Y. (2023). Effect of Different Drying Techniques on Drying Characteristics, Physical Quality, and Active Components of *Citri reticulatae pericarpium*, and the Correlation between Physiochemical Quality. Ind. Crops Prod..

[19] Peng Z., Liu Y., Zhang Y., Ai Z., Lei D., Xie Y., Wei L. (2024). Radio Frequency Roasting Promotes the Degradation of Aflatoxin B1 and Achieves Better Quality of Peanuts (*Arachis hypogaea* L.). Food Control.

[20] Zhu G., Lei D., Xie Y., Zhang Y., Shi J., Liu Y. (2024). Effects of Postharvest Piling up in Bulk on Qualities of *Camellia oleifera* Seeds. J. Stored Prod. Res..

[21] Pei Y.-P., Vidyarthi S.K., Wang J., Deng L.-Z., Wang H., Li G.-F., Zheng Z.-A., Wu M., Xiao H.-W. (2022). Effect of Vacuum-Steam Pulsed Blanching (VSPB) on Drying Characteristics and Quality Properties of Garlic Slices. Dry. Technol..

[22] Yang Y., Gao P., Sun Z., Wang H., Lu M., Liu Y., Hu J. (2023). Multistep Ahead Prediction of Temperature and Humidity in Solar Greenhouse Based on FAM-LSTM Model. Comput. Electron. Agric..

[23] Jiang D., Li C., Lin Z., Wu Y., Pei H., Zielinska M., Xiao H. (2023). Experimental and Numerical Study on the Shrinkage-Deformation of Carrot Slices during Hot Air Drying. Int. J. Agric. Biol. Eng..

[24] Ai Z., Ren H., Lin Y., Sun W., Yang Z., Zhang Y., Zhang H., Yang Z., Pandiselvam R., Liu Y. (2022). Improving Drying Efficiency and Product Quality of Stevia Rebaudiana Leaves Using Innovative Medium-and Short-Wave Infrared Drying (MSWID). Innov. Food Sci. Emerg. Technol..

[25] Perea-Flores M.J., Garibay-Febles V., Chanona-Pérez J.J., Calderón-Domínguez G., Méndez-Méndez J.V., Palacios-González E., Gutiérrez-López G.F. (2012). Mathematical Modelling of Castor Oil Seeds (*Ricinus communis*) Drying Kinetics in Fluidized Bed at High Temperatures. Ind. Crops Prod..

[26] Zhao C.-C., Ameer K., Eun J.-B. (2021). Effects of Various Drying Conditions and Methods on Drying Kinetics and Retention of Bioactive Compounds in Sliced Persimmon. LWT.

[27] Liu Z.-L., Wei Z.-Y., Vidyarthi S.K., Pan Z., Zielinska M., Deng L.-Z., Wang Q.-H., Wei Q., Xiao H.-W. (2020). Pulsed Vacuum Drying of Kiwifruit Slices and Drying Process Optimization Based on Artificial Neural Network. Dry. Technol..

[28] Liu Z.-L., Xie L., Zielinska M., Pan Z., Wang J., Deng L.-Z., Wang H., Xiao H.-W. (2021). Pulsed Vacuum Drying Enhances Drying of Blueberry by Altering Micro-, Ultrastructure and Water Status and Distribution. LWT.

[29] Yang K.-W., Wang D., Vidyarthi S.K., Li S.-B., Liu Z.-L., Wang H., Chen X.-J., Xiao H.-W. (2022). Pulsed Vacuum Drying of Persimmon Slices: Drying Kinetics, Physicochemical Properties, Microstructure and Antioxidant Capacity. Plants.

[30] Wang H., Che G., Wan L., Chen Z., Sun W., Tang H. (2023). Effect of Variable Temperature Levels on Drying Characteristics and Quality Indices of Rice in Continuous Drying and Multi-Stage Intermittent Drying. J. Food Process Eng..

[31] Li J., Huang Y., Gao M., Tie J., Wang G. (2024). Shrinkage Properties of Porous Materials during Drying: A Review. Front. Mater..

[32] Kalantari D., Naji-Tabasi S., Kaveh M., Azadbakht M., Majnooni M., Khorshidi Y., Asghari A., Khalife E. (2023). Drying Kinetics and Shrinkage Rate of Thin-Sliced Pears in Different Drying Stages. J. Food Process Eng..

[33] Zzaman W., Biswas R., Hossain M.A. (2021). Application of Immersion Pre-Treatments and Drying Temperatures to Improve the Comprehensive Quality of Pineapple (*Ananas comosus*) Slices. Heliyon.

[34] McMinn W.A.M., Magee T.R.A. (1997). Physical Characteristics of Dehydrated Potatoes—Part I. J. Food Eng..

[35] Wang N., Brennan J.G. (1995). Changes in Structure, Density and Porosity of Potato during Dehydration. J. Food Eng..

[36] Suriya M., Baranwal G., Bashir M., Reddy C.K., Haripriya S. (2016). Influence of Blanching and Drying Methods on Molecular Structure and Functional Properties of Elephant Foot Yam (*Amorphophallus paeoniifolius*) Flour. LWT Food Sci. Technol..

[37] Aradwad P.P., Thirumani Venkatesh A.K., Mani I. (2023). Infrared Drying of Apple (*Malus domestica*) Slices: Effect on Drying and Color Kinetics, Texture, Rehydration, and Microstructure. J. Food Process Eng..

[38] Ogawa T., Adachi S. (2017). Drying and Rehydration of Pasta. Dry. Technol..

[39] Gennadios A., Weller C. (1991). Edible Films and Coatings from Soymilk and Soy Protein. Cereal Foods World.

[40] Xiang Z., Ye F., Zhou Y., Wang L., Zhao G. (2018). Performance and Mechanism of an Innovative Humidity-Controlled Hot-Air Drying Method for Concentrated Starch Gels: A Case of Sweet Potato Starch Noodles. Food Chem..

[41] Liao X., Wang S., Li Y., Michael Olajide T., Zhai X., Qian J., Miao S., Huang J. (2022). Effects of “Nine Steaming Nine Sun-Drying” on Proximate Composition, Protein Structure and Volatile Compounds of Black Soybeans. Food Res. Int..

[42] dos Santos C.D., Englert A.H., Cassini A.S. (2015). Evaluation of Dispersible Protein Content During Convective Drying of Soybeans Under Different Drying Air Temperatures. Lat. Am. Appl. Res..

[43] Farmer L.J. (1996). Interactions Between Lipids and the Maillard Reaction. Flavor-Food Interactions.

[44] Aksoy A., Karasu S., Akcicek A., Kayacan S. (2019). Effects of Different Drying Methods on Drying Kinetics, Microstructure, Color, and the Rehydration Ratio of Minced Meat. Foods.

[45] An N., Sun W., Li D., Wang L., Wang Y. (2024). Effect of Microwave-Assisted Hot Air Drying on Drying Kinetics, Water Migration, Dielectric Properties, and Microstructure of Corn. Food Chem..

[46] Yao X., Zang Y., Gu J., Ding H., Niu Y., Zheng X., Zhu R., Wang Q. (2022). Microstructure Analysis and Quality Evaluation of Jujube Slices Dried by Hot Air Combined with Radio Frequency Heat Treatment at Different Drying Stages. Foods.

